# Long-term prophylaxis in hereditary angioedema: Real-world treatment patterns and healthcare resource utilization^☆^

**DOI:** 10.1016/j.waojou.2026.101399

**Published:** 2026-06-09

**Authors:** Raffi Tachdjian, Daniel F. Soteres, Maeve O'Connor, Chirag Maheshwari, Aditya Sehgal, Alice Wang, Paul K. Audhya, Timothy J. Craig

**Affiliations:** a University of California, Los Angeles, School of Medicine, Los Angeles, CA, USA; b Asthma & Allergy Associates, PC and Research Center, Colorado Springs, CO, USA; c Allergy, Asthma, & Immunology Relief of Charlotte, Charlotte, NC, USA; d Pharmsight, Haryana, India; e KalVista Pharmaceuticals, Cambridge, MA, USA; f Department of Medicine, Pediatrics, MFM and BioMedical Sciences Penn State University, Hershey, PA, USA; g Senior Advisor, Vinmec International Hospital, Hanoi, Vietnam; h Professor of Medical Sciences VinUniversity, Hanoi, Vietnam

**Keywords:** Claims data, Healthcare resource utilization, Hereditary angioedema, Long-term prophylaxis, Treatment patterns

## Abstract

**Background:**

Multiple non-androgen long-term prophylaxis (LTP) therapies have been approved in the United States to prevent hereditary angioedema (HAE) attacks. Real-world data on treatment compliance, healthcare resource utilization (HRU), and costs in this population are limited.

**Objective:**

Assess LTP treatment patterns, associated HRU, and costs in patients with HAE using a national claims database.

**Methods:**

Commercially insured patients from the IQVIA PharMetrics® Plus Closed Health Plan Claims Database (January 2016–September 2023) had ≥1 claim for non-androgen LTP, with ≥6 months of continuous enrollment pre-index and ≥12 months post-index (first LTP claim). Patient cohorts: no/minimal refill gaps, with refill gaps, or switchers. Annualized mean on-demand therapy claims, HRU, and costs were evaluated 12 months pre- and post-index.

**Results:**

A total of 328 patients were included in this analysis. Most patients (67%) had ≥1 post-index on-demand therapy claim. Mean (SD) annualized on-demand therapy doses pre- and post-LTP, respectively, were 20.8 (25.1) vs 12.4 (15.2) (*P*=0.001) with no/minimal refill gaps (n=147); 18.3 (19.7) vs 18.0 (22.3) (*P*=0.769) with refill gaps (n=131); and 25.7 (28.7) vs 29.2 (28.8) (*P*=0.12) for switchers (n=50). During follow-up, 17% and 8% had ≥1 HAE-related ER and inpatient visit, respectively. Mean annualized total HAE-related healthcare costs per patient were $165,348 pre-LTP and $515,333 post-LTP, driven by increased LTP pharmacy costs (mean $395,845 PPPY) and partially offset by reductions in medical costs (ER/inpatient, mean $8344 PPPY).

**Conclusion:**

This study found that 55% of patients had refill gaps in LTP claims, discontinued, or switched LTP within a year of initiation. Even with use of LTP, on-demand treatment utilization remained present for over two-thirds of patients. Substantial increases in total HAE-related healthcare costs were driven by LTP pharmacy costs, without significant reductions in HRU. These findings highlight the importance of monitoring and optimizing treatments for those living with HAE and ensuring access to on-demand treatment for all as the foundation for HAE management.

## Introduction

Hereditary angioedema (HAE) is a rare, autosomal-dominant genetic disease, estimated to affect 1 in 50,000–90,000 persons globally.^1^^,^^2^ HAE is characterized by unpredictable attacks of subcutaneous (SC) or mucosal swelling that may be associated with significant morbidity and can be life-threatening if the upper respiratory tract is involved.^3^

The goals of treatment in HAE are to achieve full control of the disease and to normalize patients' lives.^4^ To meet these goals, patients are recommended to have access to effective on-demand therapy, utilize short-term prophylaxis, and add long-term prophylaxis (LTP) when appropriate, taking into consideration disease activity, quality of life, availability of healthcare resources, and failure to achieve adequate control by appropriate on-demand therapy.^4^ On-demand treatment for HAE aims to reduce morbidity and prevent death during an ongoing attack.^5^ Short-term prophylaxis is used to minimize the risk of an attack in response to an expected trigger,^5^ whereas LTP is considered on an individualized basis for reducing the overall number, severity, and burden of attacks.^5^

Five non-androgen LTP treatments are approved by the United States (US) Food and Drug Administration (FDA) for patients with HAE (Supplemental Table 1).^5^^,^^6^ In the United States, approximately 70% of patients with HAE are treated with LTP, primarily non-androgens.^7^ In randomized placebo-controlled clinical trials, LTP reduced attack frequency,^8^ with attack-free rates ranging between 31% and 62% of patients.^9^ Open-label extension and real-world observational studies for LTP treatments, however, report lower attack-free rates (8%–67%), highlighting the persisting need for on-demand treatment.^9^ This difference in attack frequency may be related to LTP levels in the bloodstream falling below a minimum inhibitory concentration in less controlled environments. Indeed, efficacy is affected by LTP dosing, frequency, and PK variability.^4^ Treatment guidelines recommend regularly monitoring efficacy in patients receiving LTP and optimizing treatment outcomes by adapting dosage and treatment intervals accordingly or considering alternative approaches.^4^^,^^5^ Guidelines also acknowledge the importance of treatment compliance and recommend that expected compliance be considered prior to initiating LTP.^10^^,^^11^ Overall, successful incorporation of LTP to a patient's treatment plan requires an individualized approach with an emphasis on a high degree of monitoring and treatment compliance.^4^, ^5^, ^6^

Limited information is available on compliance with LTP treatment and the impact on healthcare resource utilization (HRU) and associated costs. Our study sought to address this gap by using a large multi-payer national administrative claims database.

## Methods

This study utilized the IQVIA PharMetrics® Plus Closed Health Plan Claims Database, which provides comprehensive real-world data on commercially insured patients in the United States. The database consists of fully adjudicated medical and pharmacy claims covering over 174 million commercially insured enrollees. The database provides a longitudinal view of inpatient and outpatient services, prescription and office/outpatient administered drugs, costs, and enrollment information. All data records were de-identified and fully compliant with US patient confidentiality requirements, including the Health Insurance Portability and Accountability Act (HIPAA) of 1996. As the study used only de-identified patient records and did not involve collection, use, or transmittal of individually identifiable data, Institutional Review Board approval to conduct the study was not necessary.

Study period was from January 1, 2016, to September 30, 2023. Index date was defined as the earliest date of the first non-androgen LTP claim during the study period. Continuous enrollment was required for at least 6 months pre-index and 12 months post-index date. Pre-index period was defined as 1 year prior to the index date and post-index was defined as 1 year after.

### Patients

Since there are no HAE-specific diagnosis codes, the study sample was identified based on HAE-exclusive medication codes. Patients with at least 1 non-androgen LTP claim during the study period were included. Patients with multiple non-androgen LTP claims on the index date and/or with an annualized claim amount more than mean ± 3 standard deviations (SD; ie, outliers) were excluded.

**Fig. 1:**
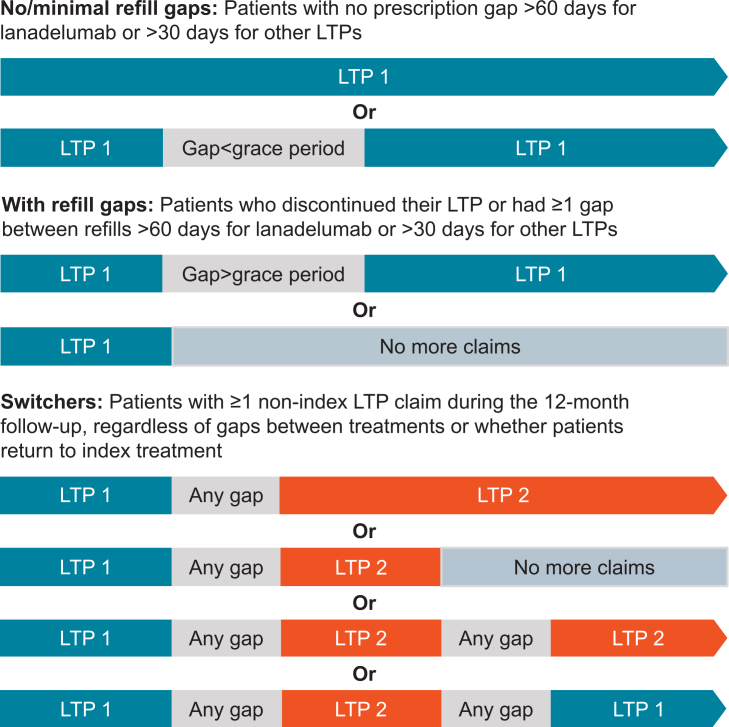
LTP patient cohorts. LTP 1 is the LTP at index date; LTP 2 is any non-index LTP. LTP: long-term prophylaxis

The study population was assessed in 3 mutually exclusive cohorts (Fig. 1): (1) no/minimal refill gaps: patients with no gaps between prescriptions that were longer than the grace period (defined below) during the 12-month post-index period; (2) with refill gaps: patients who discontinued index LTP during the post-index period or had ≥1 gap between prescription refills longer than the grace period; and (3) switchers: patients with ≥1 non-index LTP claim during the 12-month post-index period, regardless of any gaps between prescriptions or whether patients resumed index treatment after switching. Based on clinical expert opinion and FDA-approved regimens, compliant refill gaps (or grace periods) were defined as 60 days for lanadelumab and 30 days for other non-androgen LTPs, to account for varying treatment frequency indicated for different LTPs (Supplemental Table 1). Grace periods allowed for permissible gaps in refills, which could be due to common reasons, such as prescriber-directed holds, hospitalizations, or short-term use of other treatment options.^12^

### Outcomes

HRU during the pre-index and post-index period included emergency room (ER), inpatient, outpatient, and home health visits, categorized by location of care. Diagnosis and procedures for each visit were identified by International Classification of Diseases, Tenth Revision, Clinical Modification (ICD-10-CM), ICD-10 procedure coding system (ICD-10-PCS), and Current Procedural Terminology (CPT) codes. Outpatient pharmacy claims were captured by National Drug Codes (NDCs). The assessments of LTP treatment compliance included adherence (ie, no large gaps in treatment) and persistence (ie, continuous treatment). Medication adherence was measured by proportion of days covered (PDC), calculated as the percentage of days covered by index LTP prescription fills during the post-index period for the cohorts (1) with no/minimal refill gaps and (2) with refill gaps.^12^, ^13^, ^14^ The number of on-demand prescription doses was examined during pre-index and post-index periods (see Supplemental Table 2 for calculation of number of on-demand treatment doses by drug). Patients who switched or discontinued index LTP were characterized. Additional assessments included time to discontinuation and time to switch from index LTP. Associated healthcare costs, including HAE-related medical and pharmacy costs, were also assessed.

### Statistical analysis

Results are summarized as descriptive statistics (eg, mean, SD, median, interquartile range [IQR]) for continuous variables and as numbers and percentages for categorical variables. Patient demographics were assessed on the index date. Annualized mean on-demand claims, ER visits, inpatient visits, outpatient visits, home health visits, and HAE-related healthcare costs per patient per year (PPPY) were evaluated during pre-index and post-index periods. For patients with a pre-index period shorter than 364 days, these data were annualized; for patients with a pre-index period of 364 days or longer, the entire 12-month period was considered without annualization. Statistical analysis for comparison of on-demand doses by the LTP cohort was determined via a statistical package for unpaired t-test in Microsoft Excel.

## Results

### Patients

Demographics at index are described in Table 1. Overall, 328 patients were included in the analysis (Supplemental Fig. 1). Most patients were female (70%; 230/328), and the mean (SD) age at index date was 41.2 (15.5) years. Among patients on LTP, 42% (138/328) initiated Takhzyro (lanadelumab), 30% (97/328) initiated Haegarda (C1 esterase inhibitor [human]), 17% (54/328) initiated Cinryze (C1 esterase inhibitor [human]), and 12% (39/328) initiated Orladeyo (berotralstat) as the index LTP. Slightly more than half of patients (56.4%; 185/328) initiated index LTP treatment between 2016 and 2019, with the remainder (43.6%; 143/328) starting between 2020 and 2022.

**Table 1:** Demographics at index.

Parameter	All LTP	Cinryze	Haegarda	Orladeyo	Takhzyro
**Patients, n (%)**	328 (100)	54 (17)	97 (30)	39 (12)	138 (42)
**Female, n (%)**	230 (70)	42 (78)	72 (74)	25 (64)	91 (66)
**Age at index date, mean (SD)**	41.2 (15.5)	39.3 (18.3)	38.8 (15.9)	45.3 (14.5)	42.3 (14.3)
**Region, n (%)**					
South	152 (46)	23 (42)	47 (48)	14 (36)	68 (49)
Midwest	73 (22)	14 (25)	16 (16)	10 (26)	33 (24)
West	52 (16)	5 (9)	19 (20)	8 (21)	20 (15)
East	51 (16)	12 (22)	15 (15)	7 (18)	17 (12)
**Year of initiation, n (%)**					
2016	10 (3)	10 (19)	0 (0)	0 (0)	0 (0)
2017	35 (11)	26 (48)	9 (9)	0 (0)	0 (0)
2018	72 (22)	12 (22)	36 (37)	0 (0)	24 (17)
2019	68 (21)	2 (4)	19 (20)	0 (0)	47 (34)
2020	33 (10)	1 (2)	15 (15)	1 (3)	16 (12)
2021	69 (21)	2 (4)	13 (13)	23 (59)	31 (22)
2022	41 (13)	1 (2)	5 (5)	15 (38)	20 (14)
2023	N/A	N/A	N/A	N/A	N/A

LTP: long-term prophylaxis; N/A: not applicable; SD: standard deviation

### Patient cohorts

**Fig. 2:**
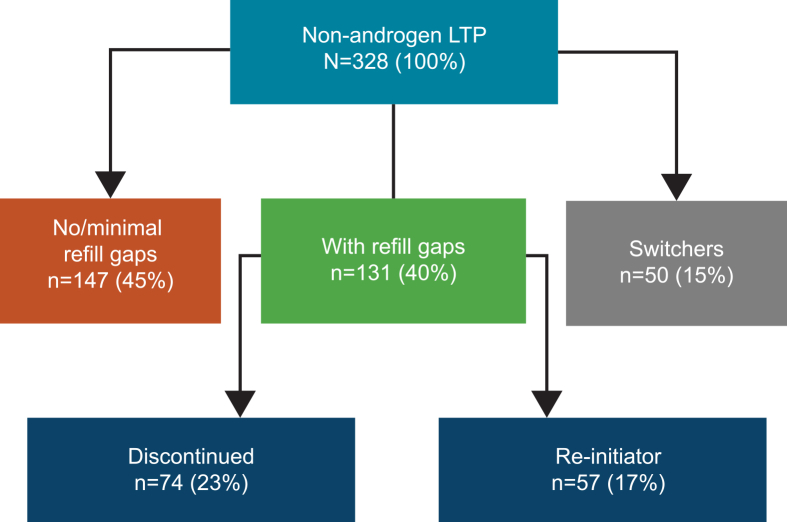
Distribution of patients by LTP cohort. LTP: long-term prophylaxis

Among the 328 enrolled patients, 45% (147/328) had no or minimal refill gaps, 40% (131/328) had refill gaps, and 15% (50/328) switched LTPs; among those with refill gaps, 56% (74/131) discontinued and 44% (57/131) re-initiated treatment (Fig. 2). Mean (SD) pre-index duration for the total LTP cohort (N=328) was 347 (40) days and a median of 364 days, with the vast majority (82%) of patients having at least a year of continuous enrollment at baseline. Mean PDC was 93% among those with no/minimal refill gaps versus 42% in those with refill gaps.

Switching patterns are summarized in Supplemental Table 3 for the 50 patients who switched LTP. The most frequent incidence of switching from the index LTP to another LTP was from Haegarda to Takhzyro (16 patients). Median (IQR) time to switch from index drug was 230 days (139–305) (Supplemental Table 4). Among the 74 patients who discontinued treatment, overall median (IQR) time to discontinuation was 53 days (28–173), with the longest median (IQR) time to discontinuation observed for Takhzyro at 94 days (28–195).

### On-demand treatment use

**Fig. 3:**
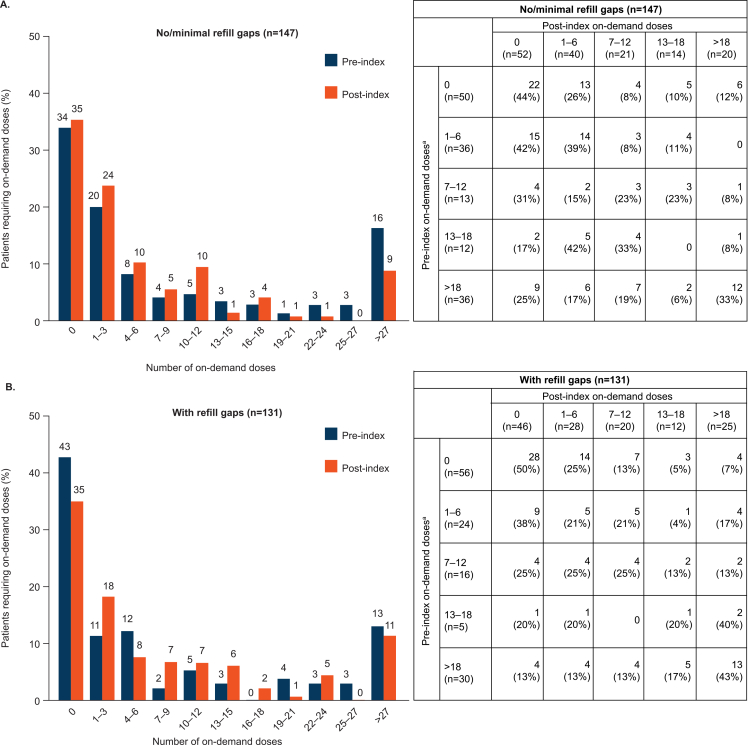
Distribution of patients with HAE by number of on-demand doses among those with no/minimal refill gaps (A) and with refill gaps (B). ^a^Percentages represent the number of patients within the post-index on-demand dose category divided by the total number of patients in the pre-index on-demand dose category. HAE: hereditary angioedema

Overall, most patients had at least 1 on-demand treatment claim both pre-index (63.1%; 207/328) and post-index (67.1%; 220/328) (Table 2). Among patients in the overall LTP cohort with at least 1 on-demand treatment claim, the mean (SD) number of on-demand doses pre-index and post-index was 20.8 (24.0) and 17.7 (21.8), respectively; the annualized median (IQR) was 11.2 (4.0–27.2) and 9.0 (3.0–20.3), respectively. Mean (SD) annualized on-demand doses were higher pre-index than post-index for those with no/minimal refill gaps (20.8 [25.1] doses vs 12.4 [15.2] doses, respectively; *P*=0.001). However, among those with refill gaps, on-demand doses remained at 18 (pre-index vs post-index: 18.3 [19.7] doses vs 18.0 [22.3] doses, respectively; *P*=0.769), whereas patients who switched LTP had a numerical increase in the number of on-demand doses (pre-index vs post-index: 25.7 [28.7] doses vs 29.2 [28.8] doses, respectively; *P*=0.12). A reduction in on-demand doses was more likely among patients with no/minimal refill gaps than those with refill gaps (odds ratio [95% CI]: 1.43 [1.24–1.65]) or among those who switched LTP therapies (odds ratio [95% CI]: 2.04 [1.60–2.60]). There was a wide distribution in proportion of patients by number of on-demand treatment doses, reflecting the high degree of heterogeneity in HAE attacks (Fig. 3). About half of the patients had ≤3 on-demand doses per year after starting LTP. The proportion of patients requiring more than 27 on-demand doses annually decreased in patients with no/minimal refill gaps (16% pre-index vs 9% post-index) after initiation of LTP compared with those with refill gaps (13% pre-index vs 11% post-index).

**Table 2:** Summary of on-demand doses pre-index vs post-index by LTP cohort.

	Number of on-demand doses per patient per year							
	Overall (N=328)		No/minimal refill gaps (n=147)		With refill gaps (n=131)		Switchers (n=50)	
	Pre-index	Post-index	Pre-index	Post-index	Pre-index	Post-index	Pre-index	Post-index
**All patients**								
Mean (SD)	13.1 (21.5)	11.8 (19.7)	13.6 (22.5)	8 (13.5)	10.5 (17.4)	11.5 (19.8)	18.5 (26.8)	23.9 (28.4)
**Patients with ≥1 on-demand dose, n (%)**	207 (63.1)	220 (67.1)	96 (65.3)	95 (64.6)	75 (57.3)	84 (64.1)	36 (72.0)	41 (82.0)
Mean (SD)	20.8 (24.0)	17.7 (21.8)	20.8 (25.1)	12.4 (15.2)	18.3 (19.7)	18.0 (22.3)	25.7 (28.7)	29.2 (28.8)

LTP: long-term prophylaxis; SD: standard deviation

### Healthcare resource utilization

During the one-year post-index period, HAE-related claims for ER and inpatient visits were observed for 17% and 8% of patients, respectively (Table 3). The proportion of patients with these types of claims was lower post-index than pre-index for those with and without (no/minimal) refill gaps. For switchers, while the proportion of patients with inpatient visits also decreased, the proportion with ER visits was higher post-index than pre-index. Most patients (81%) had claims for outpatient visits post-index. Nearly 1 in 10 LTP patients utilized at least 1 post-index home health visit, with most of the utilization associated with IV-based therapies.

**Table 3:** Summary of HAE-related healthcare resource utilization by LTP cohort.

Parameter, all patients	Overall LTP		No/minimal refill gaps		With refill gaps		Switchers	
	Pre-index (n=300)	Post-index (n=328)	Pre-index (n=138)	Post-index (n=147)	Pre-index (n=116)	Post-index (n=131)	Pre-index (n=46)	Post-index (n=50)
**No. of ER visits**								
% patients with ≥1 visit	21%	17%	18%	12%	22%	17%	26%	30%
No. visits among patients with ≥1 visit	3.1	3.4	2.1	1.8	4.4	4.4	2.6	3.9
**No. of inpatient visits**								
% patients with ≥1 visit	12%	8%	9%	5%	13%	9%	20%	10%
No. visits among patients with ≥1 visit	1.8	2.2	1.3	2.3	1.9	1.8	2.2	3.0
Length of stay, days								
Mean	3.1	4.3	2.3	5.3	4.2	4	2.4	3.5
(SD)	(2.6)	(4.8)	(1.7)	(5.5)	(3.3)	(5.1)	(1.6)	(3.3)
Median	2	3	2	3	3	2	2	3
(IQR)	(1.0, 4.0)	(1.0, 5.0)	(1.0, 3.0)	(1.0, 7.0)	(1.0, 7.0)	(1.0, 5.0)	(1.0, 3.0)	(1.0, 3.5)
**No. of home health visits**								
% patients with ≥1 visit	3%	9%	1%	5%	4%	8%	7%	20%
No. visits among patients with ≥1 visit	9.9	15.8	20.3	31.6	4.9	6	11.5	15.6
**No. of outpatient visits**								
% patients with ≥1 visit	88%	81%	88%	84%	86%	73%	89%	96%
No. visits among patients with ≥1 visit	3.2	3.9	2.9	3.3	3.7	3.7	3.3	5.8

ER: emergency room; HAE: hereditary angioedema; IQR: interquartile range; LTP: long-term prophylaxis; No.: number; SD: standard deviation

### Costs

Overall, mean annualized total HAE-related healthcare costs per patient increased from $165,348 PPPY pre-index to $515,333 PPPY post-index, primarily driven by LTP pharmacy costs (mean $395,845 PPPY), partially offset by reductions in ER/inpatient medical costs (mean −$8344 PPPY) and on-demand pharmacy costs (mean −$50,395 PPPY) (Table 4). LTP users with no/minimal refill gaps had an increase in annualized mean costs per patient (mean $431,914 PPPY), with the highest numerical increases in LTP pharmacy costs (mean $524,191 PPPY), partially offset by reductions in on-demand pharmacy costs (mean −$107,919 PPPY) and other pharmacy costs (mean −$26,282 PPPY); changes in costs of outpatient and home health visits were minimal. For the cohort with refill gaps, total healthcare costs more than doubled (mean $206,255 PPPY), with an increase in LTP costs (mean $219,900 PPPY) partially offset by a slight reduction in ER/inpatient medical costs (mean −$15,513 PPPY) and on-demand pharmacy costs (mean −$16,152 PPPY). Total healthcare costs for the switchers more than tripled (mean $487,835 PPPY), driven by increases in both LTP and on-demand pharmacy costs (mean $479,487 and $11,079 PPPY, respectively); changes in outpatient/home health and other pharmacy costs were minimal.

**Table 4:** Mean healthcare resource utilization cost per patient by cost type and by LTP cohort.

Parameter	Overall LTP		No/minimal refill gaps		With refill gaps		Switchers	
	Pre-index (n=300)	Post-index (n=328)	Pre-index (n=138)	Post-index (n=147)	Pre-index (n=116)	Post-index (n=131)	Pre-index (n=46)	Post-index (n=50)
Medical costs, ER/IP^a^	$23,060	$14,716	$10,284	$12,230	$31,159	$15,646	$31,385	$16,235
Medical costs, OP/HH/other^a^	$2255	$1668	$808	$934	$4552	$1869	$970	$3214
Pharmacy costs, on-demand^a^	$217,857	$167,462	$217,740	$109,821	$202,768	$186,616	$247,543	$258,622
Pharmacy costs, other^a^	$14,214	$4193	$26,935	$653	$4075	$7928	$2340	$1327
Pharmacy costs, LTP	$0	$395,845	$0	$524,191	$0	$219,900	$0	$479,487
Total healthcare costs	$165,348	$515,333	$165,937	$597,851	$143,843	$350,098	$217,812	$705,647

a Among patients with at least 1 claim (ie, utilized healthcare services). ER: emergency room; HAE: hereditary angioedema; HH: home health; IP: inpatient; LTP: long-term prophylaxis; OP: outpatient

## Discussion

Suboptimal adherence and persistence in chronic treatments have long been recognized as challenges to the US healthcare system.^15^ In HAE, achieving clinical success with LTP hinges on a high level of treatment compliance.^4^ This is the first longitudinal claims analysis to examine the impact of LTP compliance on changes in on-demand prescription patterns, HRU, and costs among FDA-approved non-androgen LTP treatments. Prior to the introduction of an oral LTP (ie, berotralstat), roughly one-third of patients with HAE discontinued LTP, most commonly due to the ineffectiveness of LTP and intolerable adverse effects.^16^ Our study utilized an 8-year period (2016–2023), which included the introduction of self-administered SC and oral LTP, and a label-specific definition of compliant refill gaps, altogether representing a comprehensive analysis of refill patterns for LTP and on-demand treatments in a real-world setting.

In our analysis of a moderately sized sample of 328 patients treated with currently approved LTP therapies, we assessed treatment adherence using a grace period threshold that reflected the distinction in FDA label dosing frequency between lanadelumab (60-day grace period) and other LTPs (30-day grace period). We found that 45% of patients had no or minimal refill gaps between LTP prescriptions, with overall PDC exceeding 90%. Over 1 in 5 discontinued, and among those who continued therapy, PDC was about 60%, suggesting almost 5 months out of a year without medication possession. In general, PDC greater than 80% is considered good adherence.^13^ However, in the context of HAE prophylaxis treatment regimens, 80% drug coverage means a gap of about 2.5 months in a year, which may impact the effectiveness of attack prevention. Our study also assessed post-index LTP switching, finding that a reduction in on-demand doses was less likely among patients who switched LTPs or had refill gaps, further highlighting the importance of LTP treatment compliance. It is also possible that medication switches may have occurred due to reduced efficacy.

A recent publication, Zuraw et al (2025), estimated adherence and persistence in patients treated with selected LTPs approved after 2017, including berotralstat, lanadelumab, and SC plasma-derived C1 esterase inhibitor.^17^ Compared with our study population, Zuraw et al. included a higher proportion of patients treated with berotralstat and lanadelumab, and no patients treated with IV C1 esterase inhibitor (Cinryze).^17^ Definitions for adherence and persistence measures were generally similar between our study and the Zuraw et al study, however, the latter did not assess HRU or associated costs.^17^ Zuraw et al. reported adherence rates (as measured by mean PDC) of 0.73–0.78 and 66%–69% of patients with greater than 80% PDC.^17^ These mean PDCs did not account for whether patients had refill gaps, which may explain why they fall within the range of our results: 93% for patients with no/minimal refill gaps and 42% for those with gaps. Zuraw et al. also found that 53%–61% of patients were persistent, defined as no gap in treatment ≥45 days after LTP initiation for all LTP types.^17^ We found a slightly lower proportion with no gaps or minimal gaps (ie, 45%), potentially due to the use of a regimen-specific grace period. Overall, our findings highlight that refill gaps are an important component of LTP adherence when assessing PDC, and the grace periods should be defined according to the LTP regimens.^14^

In the current study, patients utilized various HAE-related healthcare resources before initiation of LTP treatment. During the 1-year follow-up period, most (81%) had at least 1 outpatient visit claim, 17% had at least 1 ER visit claim, and 8% had at least 1 inpatient visit claim. An older US retrospective cohort study similarly found a higher proportion of patients with HAE-related claims for outpatient care (87.9%) compared with ER and inpatient visits (36.6% and 22.3%, respectively) over a 2-year period.^18^ However, that study included claims for other medications, such as androgens, fresh frozen plasma, and antifibrinolytics, whereas our analysis did not, as some of these medications may be used for other conditions. In our analysis, the proportions of patients with ER/inpatient/outpatient visits decreased overall during follow-up, but this trend was observed in the no/minimal refill gap and refill gap cohorts only, possibly indicating that inadequate efficacy with LTP leads to switching. The cohort who switched LTPs saw an increase in the proportion of patients with ER or outpatient visits, and this cohort tended to have higher proportions of patients with any type of visit. This is an unsurprising finding, considering this group may be comprised mainly of patients who have issues with LTP efficacy or tolerability due to severe or difficult-to-manage HAE symptoms and frequencies. Patients who switched LTPs or with refill gaps also had more ER visits than patients with no/minimal refill gaps, further indicating the potential importance of LTP adherence for reducing HRU.

Cost results from this study are consistent with previous reports. In this study, during the 12-month follow-up period, mean total healthcare costs PPPY were $515,333 in the overall LTP cohort, taking into account that about 40% of patients either discontinued or had large gaps between refills. Riedl et al. (2023) reported annualized total healthcare costs of over $700,000, with pharmacy costs accounting for over 95% of total costs in patients who used lanadelumab and other SC plasma-derived C1–INH LTP.^19^ Shah et al. (2023) reported mean annual HAE-specific treatment costs of over $700,000 among patients who used lanadelumab, including 46% of patients with down titration, defined as ≥25% decrease in lanadelumab costs from months 0–6 to months 7–12 or 13–18.^20^ Of note, the Shah et al. study was based on a limited sample size of 54 patients.^20^ Unlike previous studies, our analysis described HRU and costs based on LTP refill patterns. In particular, our analysis showed that patients who switched LTP treatment within the first year had the highest HRU across all locations of care, as well as on-demand treatment use and associated costs. It is also important to consider other factors affecting HRU and refill patterns. On-demand medications must be replaced when they reach their expiration date, and patients receiving LTP may be more closely followed by their healthcare providers, thereby requests to keep rescue medication available are likely more regular in this population. Additionally, over two-thirds of all patients had at least 1 on-demand treatment claim; similar rates (43%–66%) were reported in previous real-world data analyses.^20^^,^^21^ Together, these findings confirm that even with use of LTP, on-demand treatments are essential to HAE disease management.

### Limitations

Given the nature of claims database studies, there are inherent limitations to our analysis. First, continuous enrollment was required for the capture of continuous data. This resulted in the exclusion of a large proportion of patients who did not remain on the same health plan and were therefore not represented in this analysis. The characterization of LTP treatment patterns and on-demand treatment doses based on prescription medication fill claims does not reflect actual use of the medications nor the frequency, severity, or treatment status of actual HAE attacks. Notably, patients fill prescriptions for on-demand treatments prior to needing them, based on current US HAE treatment guidelines, which recommend all patients have access to on-demand medications for rapid treatment of at least 2 attacks.^5^ It is also possible for some LTP medications to be used as rescue medications, however, that information is not available in the claims database used for this analysis. Furthermore, our results may not be generalizable to all patients, such as those who are uninsured, enrolled in a public insurance plan or a clinical trial, taking free samples, part of a patient support program, or taking a prescription medication that otherwise does not need to be claimed for reimbursement, as these are not captured in this claims analysis. Other data typically not collected as part of the claims process that may be of interest for future research include indirect costs, quality of life changes, and the individual reasons for treatment gaps, switching, or discontinuation. For example, several kinds of factors can hinder treatment compliance and are unrelated to individual patients, such as socioeconomic and healthcare system-related factors.^15^ Additionally, the study sample may not geographically represent the overall US population. Another potential limitation of this study is that it identified patients with HAE based on HAE-exclusive medication codes, as there are no HAE-specific diagnosis codes; thus, the analysis did not evaluate outcomes based on different forms of HAE and may include patients with HAE with normal C1INH. Finally, our study population focused on those treated with non-androgen LTP, whereas outcomes in those treated with androgen-based LTPs were not characterized.

## Conclusion

This claims analysis showed that fewer than half of patients were adherent to LTP therapy 12 months after initiation, with more than 20% discontinuing and 15% switching. Patients with significant refill gaps had a mean PDC of 42%. Utilization of ER, inpatient, outpatient, and home health visits varied by LTP cohort, where patients with lower PDC tended to have higher HRU and costs. Total healthcare costs increased after LTP initiation, primarily driven by LTP pharmacy costs, outweighing the reductions from HRU savings even among those who were compliant to therapy. Patients who switched LTPs had the highest overall healthcare costs. Switching or discontinuing LTP may result in patient stress and uncertainty requiring additional patient support and monitoring. These findings reinforce the HAE clinical guideline recommendation of having ready access to effective on-demand treatments, which serve as the foundation of HAE disease management.^4^^,^^5^

## Abbreviations

C1INH, C1 inhibitor; Conv., conversion; CPT, Current Procedural Terminology; ER, emergency room; FDA, US Food and Drug Administration; HAE, hereditary angioedema; HCP, healthcare provider; HH, home health; HRU, healthcare resource utilization; IP, inpatient; IQR, interquartile range; IV, intravenous; LTP, long-term prophylaxis; N/A, not applicable; NDC, National Drug Code; OP, outpatient; PDC, Proportion of days covered; PPPY, per patient per year; Q, quartile; Q2W, once every 2 weeks; Q4W, once every 4 weeks; QD, once daily; SC, subcutaneous; SD, standard deviation; US, United States.

## Availability of data and materials

KalVista accepts requests from qualified researchers who wish to access clinical trial data and associated information, such as Clinical Study Reports (CSRs) with appropriately redacted appendices to protect participant privacy. Please direct your inquiry to DSP@kalvista.com for more details.

## Author contributions

AW and CM contributed to conception and design of the study. AW, CM, and AS contributed to the data analysis. All authors contributed to the interpretation of the results. All authors contributed to the review and editing of the manuscript. All authors provided final approval of the manuscript and are accountable for all aspects of the work.

## Ethics statement

The study used only de-identified patient records in full compliance with US patient confidentiality requirements, including the Health Insurance Portability and Accountability Act (HIPPA) of 1996. As the study did not involve collection, use, or transmittal of individually identifiable data, Institutional Review Board approval to conduct the study was not necessary.

## Declaration of generative AI and AI-assisted technologies in the writing process

Nothing to disclose.

## Funding source

This study was sponsored by KalVista Pharmaceuticals. KalVista Pharmaceuticals participated in the design and conduct of the study. Statistical analyses were conducted by Pharmsight and funded by KalVista Pharmaceuticals. Medical writing and editorial support for the development of this manuscript, under direction of the authors, was funded by KalVista Pharmaceuticals. Subsequent revisions and the final decision to submit the manuscript for publication were made by the authors, who vouch for the accuracy and completeness of the data and for the fidelity of the study to the protocol.

## Declaration of competing interest

**Raffi Tachdjian:** has served on advisory boards and received consulting fees/research support for Astria, BioCryst, CSL Behring, Intellia, Ionis, KalVista, Pharming, and Takeda; and had received honoraria for lectures from BioCryst, CSL Behring, KalVista, Pharming, and Takeda.

**Daniel Soteres:** has served on advisory boards for BioCryst, CSL Behring, KalVista, Pharming, and Takeda; received research support from Astria, BioCryst, Ionis, KalVista, Pharming, Pharvaris, and Takeda; and had received honoraria for lectures from BioCryst, CSL Behring, Pharming, and Takeda.

**Maeve O'Connor:** is a speaker/consultant/advisor or researcher for AbbVie, AZ, Blueprint, CSL Behring, Grifols, GSK, KalVista, Pharming, Sanofi, and Teva; and Chief Medical Officer of the CIIC.

**Chirag Maheshwari:** received consulting fees from KalVista.

**Aditya Sehgal:** received consulting fees from KalVista.

**Alice Wang:** is an employee of KalVista.

**Paul Audhya:** is an employee of KalVista.

**Timothy Craig:** received research support and was a consultant for ADARx, Astria, BioCryst, BioMarin, CSL Behring, Intellia, Ionis, KalVista, Pharvaris, and Takeda; received speaker fees from CSL Behring and Takeda; and travel support from BioCryst, CSL Behring, and Takeda.

The statements, findings, conclusions, views, and opinions contained and expressed herein are based in part on data obtained under license from the IQVIA PharMetrics® Plus Closed Health Plan database (January 1, 2016, to September 30, 2023; IQVIA Inc.). All Rights Reserved. The statements, findings, conclusions, views, and opinions contained and expressed herein are not those of IQVIA Inc. or any of its affiliated or subsidiary entities.
