# Supplementary material for: Long-term prophylaxis in hereditary angioedema: Real-world treatment patterns and healthcare resource utilization
Source: World Allergy Organ J. 2026 Jun 9;19(7):101399. doi: 10.1016/j.waojou.2026.101399 (PMC13273463; doi:10.1016/j.waojou.2026.101399)
Supplement: Multimedia component 1 [file mmc1.pdf]

Supplemental Material

Supplemental Fig. 1. Patient attrition during screening. *HAE: hereditary angioedema; LTP: long-term prophylaxis; SD: standard deviation.*

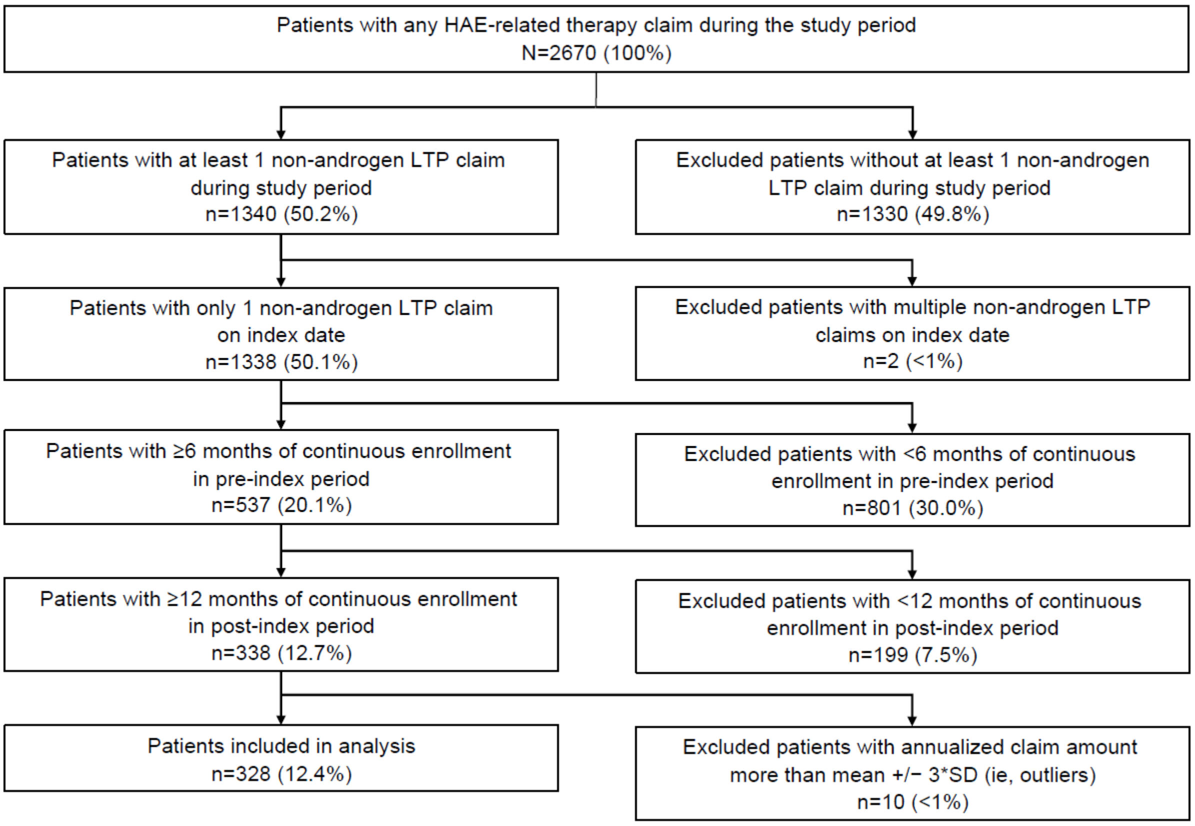

Supplemental Table 1. Current FDA-approved on-demand and LTP treatments for HAE. [a]*C1INH replacement is the preferred therapy during pregnancy and lactation.* [b]*US package insert indicates approval in adolescent and adult patients.* [c]*Cinryze is a plasma-derived C1INH replacement that is also approved for on-demand treatment of HAE attacks in patients aged ≥2 years in Europe.* [d]*Marketed as Haegarda in the US.* [e]*Product guidelines indicate use in adolescent and adult patients with HAE.* [f]*Approved after the study period.*
C1INH, C1 inhibitor; FDA, US Food and Drug Administration; HAE: hereditary angioedema; HCP: healthcare provider; IV: intravenous; LTP: long-term prophylaxis; Q2W: once every 2 weeks; Q4W: once every 4 weeks; QD: once daily; SC: subcutaneous; US: United States.

Medication	Mechanism of action	Route of administration	Indication	US approval date	Approval populations
**Berinert (C1 esterase inhibitor [human])**	C1INH replacement (plasma-derived)[a]	IV; self- or HCP-administered	On-demand (abdominal, facial, or laryngeal attacks only)	2009	US: all ages<br>Europe: all ages
**Kalbitor (ecallantide)**	Plasma kallikrein inhibitor	SC; HCP-administered	On-demand	2009	US: ≥12 yrs
**Firazyr (icatibant) – Available generically**	Bradykinin B2-receptor antagonist	SC; self-administered	On-demand	2011	US: ≥18 yrs<br>Europe: ≥2 yrs
**Ruconest (C1 esterase inhibitor [recombinant])**	C1INH replacement (recombinant)[a]	IV; self- or HCP-administered	On-demand	2014	US: ≥12 yrs[b]<br>Europe: ≥2 yrs
**Cinryze (C1 esterase inhibitor [human])**	C1INH replacement (plasma-derived)[c]	IV; self-administered	LTP<br>(Every 3-4 days, 1000 IU; 500 IU in children 6-11 yrs old)	2008	US: ≥6 yrs<br>Europe: ≥6 yrs
**Berinert 2000/3000 (Haegarda, C1 esterase inhibitor [human])[d]**	C1INH replacement (plasma-derived)	SC; self-administered	LTP<br>(Twice weekly, 60 IU/kg)	2017	US: ≥6 yrs<br>Europe: ≥12[e] yrs
**Takhzyro (lanadelumab)**	Plasma kallikrein inhibitor	SC; self-administered	LTP<br>(Q2W or Q4W, 300 mg; 150 mg Q2W [age 6 to <12 yrs]; 150 mg Q4W [age 2 to <6 yrs])	2018	US: ≥2 yrs<br>Europe: ≥2 yrs

**Orladeyo (berotralstat)**	Plasma kallikrein inhibitor	Oral; self-administered	LTP<br>(QD 150 mg; 110 mg reduced dose)	2020	US: ≥12 yrs<br>Europe: ≥12 yrs
**Andembry (garadacimab-gxii)**[f]	Factor XII inhibitor (monoclonal antibody)	SC; self-administered	LTP<br>(Initial loading dose of 400 mg; once monthly, 200 mg)	2025	US: ≥12 yrs<br>Europe: ≥12 yrs

Supplemental Table 2. Calculation of number of on-demand treatment doses. *An example calculation is as follows: A patient has two claims for Ruconest: one with the NDC code for Ruconest and the other with the CPT (procedure) code for Ruconest. The NDC Code: Quantity = 8; Procedure Code: Quantity = 420; Conversion to Eaches (IU): NDC: 8/1 = 8 eaches; Procedure: 420/210 = 2 eaches; Total IU: 8 + 2 = 10 eaches. The conversion of Eaches to doses/attacks: 10/2 = 5 doses. Thus, the total number of Ruconest doses is 5.*
Conv.: conversion; CPT: Current Procedural Terminology; NDC: National Drug Code.

Medication	Based on NDC code		Based on procedure code			Conversion
	Unit	Conv. to eaches	Unit	Conv. to eaches	Per vial/syringe	Eaches to doses
Berinert	Vial	Divide by 1	10 IU	Divide by 50	500 IU	Divide by 4
Ruconest	Vial	Divide by 1	10 IU	Divide by 210	2100 IU	Divide by 2
Kalbitor	Vial	Divide by 1	1 mg	Divide by 10	10 mg	Divide by 3
Icatibant	mL	Divide by 3	1 mg	Divide by 30	30 mg	Divide by 1

Supplemental Table 3. Overview of LTP change patterns in the switcher cohort.
LTP: long-term prophylaxis.

Index drug	Switch drug(s)	Counts
Cinryze	Haegarda	9
Cinryze	Haegarda and Takhzyro	3
Cinryze	Takhzyro	2
Haegarda	Cinryze	1
Haegarda	Orladeyo	2
Haegarda	Takhzyro	16
Orladeyo	Haegarda	5
Orladeyo	Takhzyro	2
Takhzyro	Cinryze	2
Takhzyro	Haegarda	3
Takhzyro	Orladeyo	5

Supplemental Table 4. Time to switch from index LTP drug in the switcher cohort.
LTP: long-term prophylaxis; SD: standard deviation; Q: quartile.

	**Time to switch, days**					
**Switcher cohort**	**Patients**	**Mean**	**SD**	**Median**	**Q1**	**Q3**
All	50	221	95	230	139	305
Cinryze	14	229	97	247	174	304
Haegarda	19	227	90	252	137	303
Orladeyo	7	220	90	207	142	290
Takhzyro	10	201	112	200	113	284
